# The Role of *TRPM4* Gene Mutations in Causing Familial Progressive Cardiac Conduction Disease: A Further Contribution

**DOI:** 10.3390/genes13020258

**Published:** 2022-01-28

**Authors:** Alberto Palladino, Andrea Antonio Papa, Roberta Petillo, Marianna Scutifero, Salvatore Morra, Luigia Passamano, Vincenzo Nigro, Luisa Politano

**Affiliations:** 1 Cardiomiology and Medical Genetics, University Hospital of Campania Luigi Vanvitelli, 80138 Naples, Italy; alberto.palladino@libero.it (A.P.); roberta.petillo@live.it (R.P.); scutifero.marianna59@libero.it (M.S.); salv.morra@gmail.com (S.M.); luigia.passamano@unicampania.it (L.P.); 2 Division of Cardiology, Department of Translational Medical Sciences, University of Campania “Luigi Vanvitelli”, Monaldi Hospital, 80131 Naples, Italy; andreaantonio.papa@libero.it; 3 Department of Precision Medicine, University of Campania Luigi Vanvitelli, 80138 Naples, Italy; vincenzo.nigro@unicampania.it

**Keywords:** progressive cardiac conduction disease, atrio-ventricular block, right bundle branch block, Cardiac channelopathy

## Abstract

Progressive cardiac conduction disease (PCCD) is a relatively common condition in young and elderly populations, related to rare mutations in several genes, including *SCN5A, SCN1B, LMNA* and *GJA5, TRPM4.* Familial cases have also been reported. We describe a family with a large number of individuals necessitating pacemaker implantation, likely due to varying degrees of PCCD. The proband is a 47-year-old-patient, whose younger brother died at 25 years of unexplained sudden cardiac death. Three paternal uncles needed a pacemaker (PM) implantation between 40 and 65 years for unspecified causes. At the age of 42, he was implanted with a PM for two episodes of syncope and the presence of complete atrioventricular block (AVB). NGS analysis revealed the missense variation c. 2351G>A, p.Gly844Asp in the exon 17 of the TRPM4 gene. This gene encodes the TRPM4 channel, a calcium-activated nonselective cation channel of the transient receptor potential melastatin (TRPM) ion channel family. Variations in *TRPM4* have been shown to cause an increase in cell surface current density, which results in a gain of gene function. Our report broadens and supports the causative role of *TRPM4* gene mutations in PCCD. Genetic screening and identification of the causal mutation are critical for risk stratification and family counselling.

## 1. Introduction

Progressive cardiac conduction disorder (PCCD) is an inherited cardiac disease that may present as a primary electrical disease or in association with structural heart diseases, and often requires pacemaker (PM) implantation [1]. Because PM implantation improves survival [2], conduction disease poses an important health problem. Causal loci have been mapped for single-gene diseases involving atrioventricular conduction in isolation or associated with cardiomyopathy or skeletal myopathy; for a number of diseases, a causative gene has been identified [3]. 

The pathogenesis of the inherited PCCD in structurally normal hearts was linked to genetic variants in the ion channel genes *SCN5A, SCN1B, SCN10A, TRPM4* and *KCNK17* as well as in genes coding for cardiac connexin proteins, such as *GJA5*, associated with lone atrial fibrillation. [4]. *LMNA* mutations are also frequently reported in patients with life-threatening ventricular arrhythmias and significant cardiac conduction disorders, even if left ventricular ejection fraction is preserved [5]. Genetic forms of PCCD can often overlap or coexist with other inherited heart diseases or manifest in the context of multisystem syndromes. 

Other genes coding for cardiac transcription factors, such as *NKX2.5* and *TBX5,* may be involved in the development of a cardiac conduction system and in morphogenesis of the heart [6]. Mutations in the two above mentioned genes may cause cardiac conduction disorders associated with various congenital heart defects. 

However, despite the significant advances in the knowledge of the genetic architecture of PCCD and overlapping diseases, in a measurable fraction of cases, including in familial clustering of disease, investigations of known cardiac disease-associated genes fail to reveal the underlying substrate, suggesting that new causal genes are yet to be discovered. 

A precise genetic diagnosis is essential for risk stratification and better selection of specific therapy; it also allows familiar cascade screening and appropriate genetic counseling. Cardiologists should be aware of the different phenotypes emerging from different gene-mutations and the potential risk of sudden cardiac death. 

Isolated cardiac conduction blocks are not infrequent in the general population, prevalent in about 0.5% of children and occurring more in the 6th decade and above, according to recent surveys [6].

**Figure 1:**
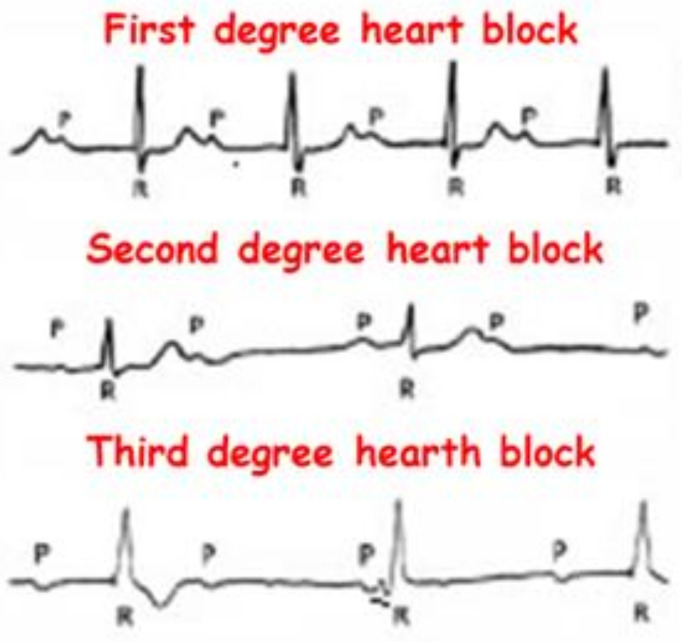
A schematic example of 1st, 2nd and 3rd degree AV block is shown as they appear on a 12-lead ECG.

Atrioventricular block (AVB) is the partial (1st or 2nd degree AVB) or complete (3rd degree AVB) interruption of the transmission of the electrical impulse from the atria to the ventricles (Figure 1). The most frequent causes are fibrosis and idiopathic sclerosis of the conduction system. Diagnosis is based on the ECG. Symptoms and therapy depend on the degree of blockage, but treatment, when needed, usually involves implantation of a pacemaker [7].

In progressive familial heart block type I (PFHBI; also known as PFHBIB), an autosomal dominant inherited disease of the His-Purkinje system, disease progression is initially characterized by the occurrence of right bundle branch block (RBBB), followed by bi-fascicular block and finally by complete heart block (CHB) [8]. PFHBI was mapped to the kallikrein I [KLK1] locus, on chromosome 19q33 [9], but was also associated with mutations in ion channel genes *SCN5A* [10]. PFHBII differs clinically from PFHBI in that the CHB occurrence is consistent with an atrioventricular nodal level disease [9], rather than a ventricular conduction disease. Kruse et al [11], further refining the genetic interval for the PFHBI disease locus, showed that the missense mutation c.19G→A in *TRPM4* gene was the cause of blunted cardiac conduction in several branches of a large Afrikaner family they described. 

Transient receptor potential channel melastatin 4 or TRPM4 is a Ca2+ activated non-specific cationic channel, impermeable to Ca2+, which is part of the transient receptor potential channel (TRP) family [12,13]. *TRPM4* in particular is expressed in cardiac cells of the conduction pathway and arterial and venous smooth muscle cells [14,15,16,17,18]. Genetic variants in the human gene *TRPM4* have been associated with several cardiac conduction disorders. Hypertensive rats with cardiac hypertrophy have been shown to spontaneously over-express the cardiac TRPM4 channel [19]. Dominantly inherited mutations in the *TRPM4* gene are associated with PFHBI and isolated cardiac conduction disease (ICCD), giving rise to the atrio-ventricular conduction block (AVB), right bundle branch block (RBBB), bradycardia, and Brugada syndrome. Demion et al. [19] investigated the role of the TRPM4 channel on whole cardiac function on a *TRPM4* gene knock-out mouse (Trpm4-/-) model. Morpho-functional analysis revealed in these mice a left ventricular (LV) eccentric hypertrophy, with an increase in both wall thickness and chamber size in adult mice aged 32 weeks when compared to Trpm4+/+ littermate controls. Furthermore, Trpm4-/- mice presented multilevel conduction blocks, as attested by PR and QRS lengthening in surface ECGs and confirmed by intra-cardiac exploration. As a nonselective monovalent cation channel, *TRPM4* upregulation and activation enhance sodium entry, which leads to depolarization of the membrane potential. The membrane potential is critical in regulating calcium influx; calcium dysregulation is known to play an essential role in predisposing to tachy-arrhythmias and sudden cardiac death [20,21,22].

In the present study, we describe a family from South Italy with a large number of individuals necessitating pacemaker implantation likely due to varying degrees of PCCD, transmitted in an autosomal dominant manner and incomplete penetrance.

## 2. Patients and Methods

Family history. Parents unrelated, both with high blood pressure. On the paternal side, three uncles needed a pacemaker (PM) implant between the age of 40 and 65 years, for unspecified causes. The three uncles of the patient were dead at the time of the study and no clinical information was available for two of them. Only for the youngest uncle did the patient report that he was diagnosed with extreme bradycardia and high risk of cardiac arrest during a cardiologic check-up for dyspnea, and was immediately implanted. He was hypertensive.

**Figure 2:**
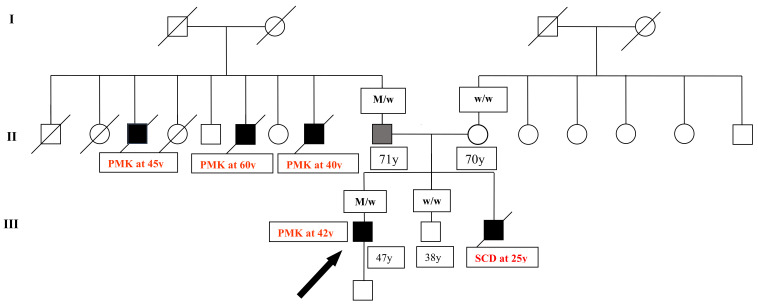
Pedigree of the family. Family members are identified by generations. Squares = males; circles = females; black symbols = affected individuals; white symbols = unaffected individuals; grey symbol = individual carrying *TRPM4* gene mutation, without AVB; arrow = the proband; M/w = people carried *TRPM4* mutation; w/w = people without *TRPM4* gene mutation.

The proband, currently 47, is the first of three siblings, all males, the last of whom died in his sleep at the age of 25 (Figure 2). 

Personal history. Born at term from operative delivery (cesarean), the developmental milestones were normal. A cardiological examination performed at 10 years led to the diagnosis of incomplete right bundle branch block (RBBBi) for which he was exempted from military service. Despite this, from 7 to 33, he played football, first as a player, then as a coach. Two surgeries at 10 and 14 years, respectively, of appendectomy and rhinoseptoplasty were performed under general anesthesia, without complications. 

At the age of 36, he presented a first episode of syncope, which has not been further investigated. After 6 years, a new episode of syncope required access to the local emergency room. On that occasion, he was diagnosed with complete atrio-ventricular block (AVB) on the ECG, for which he was implanted with a dual chamber pacemaker; he was discharged without cardiological treatment. A modest increase in serum creatinkinase (CK) levels was also observed (303 U/L vs. 170 U/L). 

**Figure 3:**
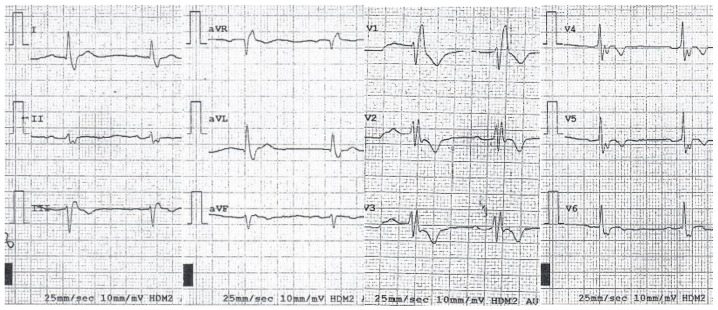
Proband’s ECG tracing showing an incomplete right bundle branch block (RBBBi) associated with 1st degree atrio-ventricular block (AVB).

Due to the concomitance of increased values serum CK, family history of sudden cardiac death and pacemaker implantation, the patient was advised to contact the Monaldi Hospital in Naples, and, from there, was sent to our service. The last ECG performed before admission to our service showed a first-degree AVB associated with a complete RBBB (Figure 3), while the echocardiogram showed a left ventricle ejection fraction (LVEF) of 45%. 

He started therapy with beta-blockers and ACE-inhibitors. Upon admission, the patient denied angor, palpitations and muscle symptoms, but complained of exertional dyspnea, even when walking. 

Neuromuscular examination showed normal strength at girdle and distal lower limbs (deltoids, biceps and triceps brachialis 5/5, tibialis anterior e peroneal muscles 5/5), pes cavus, lumbar hyperlordosis, and normal deep tendon reflexes. 

**Figure 4:**
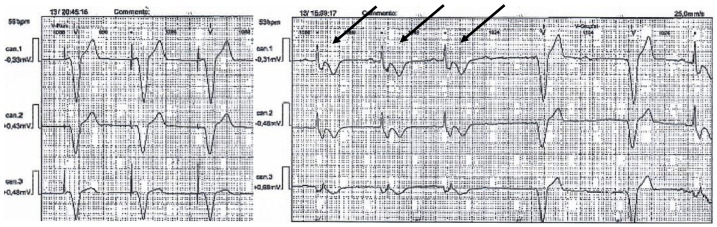
Proband’s dynamic ECG tracing showing PM rhythm with sporadic episodes of spontaneous rhythm (arrows).

Cardiological examination revealed pure, rhythmic heart tones and a systolic murmur in the left fourth intercostal space. The ECG showed a PM rhythm, with a mean ventricular response of 56 beats/min. Dynamic ECG showed a PM rhythm with sporadic episodes of spontaneous rhythm, and repolarization anomalies when the PR interval was normal; during the PM rhythm, however, a first degree AVB appeared with a widened, electro-induced QRS interval (Figure 4).

Echocardiogram showed dilated hypokinetic cardiomyopathy, with reduced LVEF (35%). Respiratory function was normal. 

Pharmacological treatment was enhanced with the addition of diuretics (furosemide and potassium canrenoate), and folic acid for the detection of hyperhomocysteinemia. 

At the last check-up, at age 47, the patient reported well-being, denied palpitations, exertional dyspnea or tachycardia. No muscular and respiratory symptoms. PM checks every six months. 

The cardiological examination remained unchanged. ECG showed sinus rhythm, with first degree AVB (PR interval = 240 msec) and complete RBBB. Echocardiogram confirmed the presence of dilated cardiomyopathy, but LVEF was improved to 62%. Right heart chambers were of normal size and kinetics.

**Figure 5:**
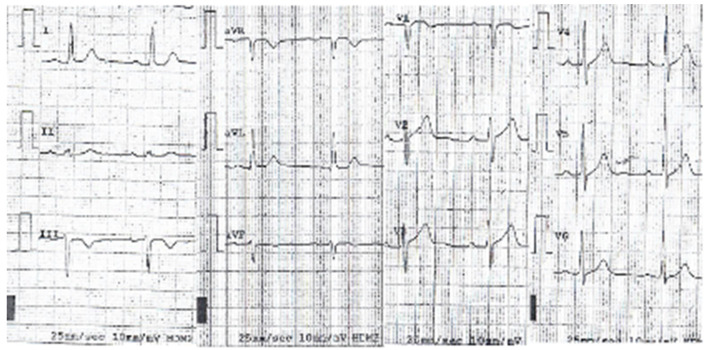
Proband’s father ECG tracing showing a normal sinus rhythm, without signs of atrio-ventricular or bundle branch block.

The proband’s father was also cardiologically evaluated. With the exception of arterial hypertension in drug treatment for years, no other pathology or specific symptomatology was reported. The ECG was normal (Figure 5). 

A signed informed consent was obtained from the patient and his family prior to recording and blood drawing for DNA analysis. The study was conducted in accordance with the Declaration of Helsinki.

### Mutation Screening

Genomic DNA was extracted from peripheral blood in the patient, his parents and the brother, by using standard procedures. For the mutation screening, the NGS technology was used. The panel included 2742 genes, all of which are involved in mendelian diseases, in muscular dystrophies and, in particular, those more frequently associated with cardiac conduction disturbances, arrhythmias and dilated cardiomyopathy, and enriched with Sure Select technology and Agilent Technology (Inherited Disease Panel). Parents and brother were also included in the NGS study. 

## 3. Results

We first focused on screening for mutations in *LMNA*, desmin, myotilin and *LDB3* genes because, in our experience, mutations in these genes are seen more frequently in patients with cardiac conduction defects and increased CK values. These genes showed no mutations. NGS analysis discovered the single heterozygous mutation c. 2351 G>A; p.Gly844Asp in exon 17 of the *TRPM4* gene. The father also showed this mutation, while the mother and the unaffected brother did not.

## 4. Discussion

In this report, a proband with 1st degree AVB associated with complete RBBB and a history of syncope was investigated by NGS analysis. The missense mutation *TRPM4* c.2351G>A; p.Gly844Asp, which is located in the exon 17, was identified. This missense mutation was further confirmed in other family members by Sanger sequencing, suggesting that it co-segregates with the disease phenotype, and is likely responsible for PCCD in this family. The TRPM4 channel plays a crucial role in the cardiac conduction system. Immunohistochemistry results have shown that TRPM4 is highly enriched in ventricular cardiomyocytes and is highest in Purkinje fibers [23]. In previous studies, Mathar et al. demonstrated that Trpm4 knock-out mice have a shortened ventricular action potential and an increased β-adrenergic-dependent ventricular systole [24]. In 2015, Jacobs et al. showed that deletion of the TRPM4 gene in mice improves survival and significantly enhances β-adrenergic cardiac reserve after inducing ischemic HF [25]. In 2009, Kruse et al. identified the first *TRPM4* gene mutation in PFHBI patients [11]. Very recently, Dong et al. reported a novel *TRPM4* mutation in a Chinese Family with atrioventricular block [26], supporting the idea that *TRPM4* may act as a major gene predisposing to progressive familial heart block type I [27]. 

So far, 25 TRPM4 gene mutations have been reported in the Human Gene Mutation database at https://digitalinsights.qiagen.com/gene_mutation/database (accessed on 28 December 2021). Twenty-four of them are missense/nonsense mutations and one, a small insertion. Of these 25 mutations, 9 were associated to isolated cardiac conduction disease [11,27,28,29], 8 + 1 (?) to Brugada syndrome [30,31], 3 to long QT syndrome [32], 2 to type 1 AVB [11,27] and 1 to unexpected sudden death in infancy [33] (Table 1). 

The Gly844Asp variant found in our family had already been reported in multiple individuals, in association with cardiac conduction disorders [27,28,29,30,31]. The 2531 G-A transition in exon 17 resulting in Gly844Asp substitution occurs at a conserved residue within an intracellular sequence connecting the second and third transmembrane segments [28]. 

Liu et al [28] found this variation in several family members with incomplete RBBB or no block, consistent with incomplete penetrance. However, they did not find it in 300 ethnically matched control chromosomes. Furthermore, by functional analysis in HEK293 cells, Kruse et al. [11] demonstrated that current amplitudes were dramatically elevated for mutant versus wild type channels, despite no increase in Ca2+ affinity for the mutant channels compared to wild type. By quantitative analysis of transfected COS-7 cells, they showed that the gain of function was due to increased density of mutant channels at the cell surface, which, in turn, was related to impaired endocytosis and deregulation of SUMOylation.

The same variation was identified by Stallmeyer et al. [29] in two unrelated German patients with RBBB and left anterior hemiblock (LAHB). One patient was an asymptomatic 11-year-old boy with RBBB and LAHB but a normal heart rate; his 45-year-old mother, who had a normal electrocardiogram (ECG), also carried the mutation. The other patient was a 17-year-old boy who was compound heterozygous for Gly844Asp and a polymorphic in-frame deletion (2283-2294del, resulting in R762-G765del); his ECG showed a ‘bizarre and obvious’ RBBB and LAHB. His 45-year-old father, who also carried the mutation, had a normal ECG, as did his 44-year-old mother, who was a heterozygous carrier of the polymorphism.

However, the pathogenicity of this variation remains controversial, because several of these probands harbored additional cardiogenetic variants, and some of the relatives who were heterozygous for Gly844Asp were unaffected at the time of the examination [28,29,34,35,36]. Nevertheless, Gly844Asp is a non-conservative amino acid substitution, which can affect the secondary protein structure, as these residues differ in polarity, charge, size and/or other properties. This variant was described as probably damaging (0.945) with PPH-2 by Daumy et al. [27], in a homozygous state in a patient presenting with RBBB and left anterior hemiblock (LAHB) on ECG, and phenotypic AVB 2/1, 3/1. Furthermore, as previously indicated, functional studies suggest that the Gly844Asp variant causes a gain-of-function effect due to an elevated TRPM4 channel density at the cell surface [28]. Recently, an altered protein stability caused by *TRPM4* cation channel mutations has been reported by Bianchi et al. [34]. Variants in the TRPM4 gene were associated with either gain- or loss-of-function of TRPM4 channels for a similar clinical phenotype. These findings plead in favor of the implication of multiple factors rather than simple TRPM4 gain- or loss-of-function to cause diseases [35,37].

However, our study, in combination with previously published papers, strongly support the prominent role of this cardiac TRP channel in this subtype of conduction disease. The clinical onset of conduction disturbances tends to occur at an early age among affected patients, as bundle branch blocks, and evolve in median/old age towards atrio-ventricular blocks. In this study, the presence of four cases implanted with a PM at varying age and early sudden cardiac death in a fourth family member suggest an important role of inheritance in disease severity. Furthermore, the observation that the proband’s father, despite the mutation, does not have AVB, also suggests that additional genetic factors may determine the susceptibility to the disease.

## Figures and Tables

**Table 1:** List of *TRPM4* mutations so far reported in human gene mutation database.

Phenotype	Codon Change	Amino Acid Change	Protein	Reference of First Description
Brugada Syndrome	GGG-AGG	Gly-Arg	Gly555Arg	Liu, 2013 [30]
	TTC-ATC	Phe-Ile	Phe773Ile	Liu, 2013 [30]
	CCG-CGG	Pro-Arg	Pro779Arg	Liu, 2013 [30]
	CAG-CGG	Gln-Arg	Gln854Arg	Liu, 2013 [30]
	ACC-ATC	Thr-Ile	Thr873Ile	Liu, 2013 [30]
	AAA-TAA	Lys-Term	Lys914Term	Liu, 2013 [30]
	CTG-CCG	Leu-Pro	Leu1075Pro	Liu, 2013 [30]
	CCG-CTG	Pro-Leu	Pro1204Leu	Liu, 2013 [30]
Brugada Syndrome (?)	CGG-TGG	Arg-Trp	Arg144Trp	Liu, 2013 [30]
Cardiac Conduction Disease	CAG-CAC	Gln-His	Gln131His	Stallmeyer, 2012 [29]
	CGG-TGG	Arg-Trp	Arg164Trp	Liu, 2013 [30]
	CAG-CGG	Gln-Arg	Gln293Arg	Stallmeyer, 2012 [29]
	GCC-ACC	Ala-Thr	Ala432Thr	Liu, 2010 [28]
	GGT-AGT	Gly-Ser	Gly582Ser	Stallmeyer, 2012 [29]
	TAC-CAC	Tyr-His	Tyr790His	Stallmeyer, 2012 [29]
	GGC-GAC	Gly-Asp	Gly844Asp	Liu, 2010 [28]
	AAA-AGA	Lys-Arg	Lys914Arg	Stallmeyer, 2012 [29]
	CCC-TCC	Pro-Ser	Pro970Ser	Stallmeyer, 2012 [29]
Heart Block Type 1	GAG-AAG	Glu- Lys	Glu7Lys	Kruse, 2009 [11]
	ATA- ACA	Ile-Thr	Ile376Thr	Daumy, 2016 [27]
Long QT Syndrome	CTG-ATG	Val-Met	Val441Met	Hof, 2017 [32]
	CGG-CCG	Arg-Pro	Arg499Pro	Hof, 2017 [32]
	CGG-TGG	Arg-Trp	Arg499Trp	Hof, 2017 [32]
Sudden unexpected death in infancy	TGG-TGA	Trp-Term	Trp5252Term	Hertz, 2016 [33]
